# Digital loneliness as a new diagnostic category in psychiatry: the impact of technology and social media use on psychological well-being

**DOI:** 10.3389/fpsyt.2025.1630275

**Published:** 2025-08-28

**Authors:** Daniel Szawarnoga, Artur Stasiniewicz, Joanna Fojcik, Marek Krzystanek

**Affiliations:** ^1^ Department of Psychiatry, Department of Neurology, Faculty of Health Sciences in Katowice, Medical University of Silesia in Katowice, Poland; ^2^ Department of Neurology, Faculty of Medical Sciences in Zabrze, Medical University of Silesia in Katowice, Poland; ^3^ Department and Clinic of Psychiatric Rehabilitation in Katowice, Faculty of Medical Sciences in Katowice, Medical University of Silesia in Katowice, Poland

**Keywords:** digital loneliness, social media, loneliness, anxiety symptoms, digital hygiene, mental health, education

## Abstract

**Purpose:**

The aim of this study was to determine the impact of social media use on users’ subjective feelings of loneliness and the occurrence of anxiety symptoms, taking into account demographic variables such as age and education level.

**Material and methods:**

A total of 220 patients of the Mental Health Outpatient Clinic participated in the study and completed a self-administered survey questionnaire including demographic data, social media use patterns and assessment of experienced psychological symptoms.

**Results:**

Statistical analyses showed that younger users spend more time online, which correlates with higher feelings of loneliness and severity of anxiety symptoms, while higher levels of education are associated with less time online and better awareness of the dangers of excessive social media use.

**Conclusions:**

Social media leads to an increased sense of isolation. Excessive use of these platforms is associated with decreased sleep quality, decreased self-esteem, increased levels of stress and anxiety and an overall deterioration in users’ mental health. The experience of digital loneliness varies according to the characteristics of users - young people, women and people with limitations are the most vulnerable to the negative effects of excessive social media use.

## Introduction

With over 25 years of social media existence, one might be tempted to say that the field of social media and its impact on various elements of human life is very well researched. However, this statement would be untrue, as social media is constantly evolving, constantly changing. Therefore, their impact on human life is variable over time. The social media environment is evolving so rapidly that some of the scientific research that was conducted a few or several years ago should be considered outdated.

Using social media (and especially social networking sites) is nowadays possible practically anywhere and anytime. All that is needed to connect to the internet is: a smartphone, mobile phone network coverage and a purchased mobile internet package or Wi-Fi access. The indicated requirements are so low that most young people (in developed and developing countries) have constant access to the Internet via their mobile devices. This, in turn, means that young people are usually ‘plugged in’ to the internet all day long, and respond immediately to any new message coming from social media.

Originally designed as a tool to facilitate communication, social media have become an integral part of modern life. Their impact on psychological well-being, especially in the context of young people and the elderly, is of increasing interest to researchers (1, 2). In the literature we find both positive and negative aspects of online functioning. Excessive use of social media is associated with, among other things, lowered self-esteem (3) and a phenomenon referred to as ‘Facebook introversion’ (4), while reduced time spent online correlates with better emotional functioning (5, 6). Contemporary research points to the complexity of the relationship between technology and mental health, particularly in the context of excessive social media activity.

This thesis focuses on the analysis of digital loneliness as a phenomenon with potential clinical and social relevance, in the context of rapidly evolving technologies. Among other things, the long-term effects of intensive social media use on mental health, self-esteem and the quality of interpersonal relationships were examined. A review of numerous empirical and psychosocial studies provided a critical look at existing scientific evidence and the identification of research gaps.

## Materials and methods

The main aim of the study was to determine how intensive social media use affects users’ mental health (particularly feelings of loneliness and anxiety symptoms), taking into account demographic variables such as age and education level, as well as digital loneliness as an important element of this process. The research question of the potential of digital loneliness as a new diagnostic category is also addressed, in light of the increasing number of people reporting problems of social isolation caused by technology.

A total of 220 patients of the Mental Health Outpatient Clinic in Bytom took part in the study. The group was diverse in terms of age, education and level of social media activity. Adults aged 35–54 years were the largest group (58%), while those under 24 years of age accounted for about 9%. The majority of participants had a secondary or tertiary education. They were patients reporting emotional difficulties, in whom anxiety symptoms, feelings of loneliness and social media use patterns, among others, were assessed.

The detailed age structure of the respondents was as follows:

- Under 18 years: 3,6%
- 18–24 years: 5,5%
- 25–34 years: 18%
- 35–44 years: 28%
- 45–54 years: 30%
- 55 years and over: 15%

A proprietary survey questionnaire was used for the study, which consisted of three main parts:

1. Demographics: age, gender, education level.
2. Social media usage patterns: frequency of use, average daily hours and preferred platforms (with a focus on Facebook).
3. Assessment of psychological symptoms experienced: subjective assessment of feelings of loneliness and the presence of anxiety and depressed mood symptoms.

Statistical analyses were performed using IBM SPSS Statistics (version 26.0). The chi-square test and effect measures such as Cramér’s Phi and V coefficients, as well as Kendall’s Tau-b and Tau-c coefficients for the assessment of rank correlations were used in the dependency analysis.

The choice of statistical methods, such as Kendall’s Tau-b and Tau-c coefficients, was dictated by the nature of the data - ordinal and rank variables. A similar approach has been used in studies on the relationship between social media use and mental state (7, 8) which confirms the appropriateness of the analyses used.

## Results

The analysis showed that age significantly influenced the frequency of social media use - older respondents used social media less frequently than younger respondents (τc = -0.339, p < 0.001). In addition, a significant negative correlation was observed between education level and time spent online - those with higher education spent less time online.

Respondents who spent more hours online reported higher levels of perceived loneliness (τb = 0.277, p < 0.01).

Additionally, younger users were more likely to experience anxiety symptoms, as confirmed by statistical analyses (τc = -0.116, p < 0.05) [8]. These findings are in line with previous research on the impact of excessive social media use on mental status, particularly among adolescents.

The analysis revealed that higher levels of education are associated with greater awareness of the risks of heavy social media use (τc = 0.110, p < 0.05). The literature highlights that such awareness is an important element in the prevention of digital addictions. The results are presented in the **Tables 1**–**4**.

**Table 1:** Patterns of social media use and demographic variables.

Tested variables			Age						Total
			Under 18 years	18–24 years	25–34 years	35–44 years	45–54 years	55 years and over	
How many hours a day on average do you spend on social media?	Less than 1 hour	N	0	0	3	14	23	17	57
		%	0,0%	0,0%	7,5%	22,6%	34,8%	53,1%	25,9%
	1–2 hours	N	1	2	20	24	29	12	88
		%	12,5%	16,7%	50,0%	38,7%	43,9%	37,5%	40,0%
	2–4 hours	N	4	8	12	18	11	1	54
		%	50,0%	66,7%	30,0%	29,0%	16,7%	3,1%	24,5%
	4–6 hours	N	3	1	4	4	3	2	17
		%	37,5%	8,3%	10,0%	6,5%	4,5%	6,3%	7,7%
	Over 6 hours	N	0	1	1	2	0	0	4
		%	0,0%	8,3%	2,5%	3,2%	0,0%	0,0%	1,8%
Total		N	8	12	40	62	66	32	220
		%	100,0%	100,0%	100,0%	100,0%	100,0%	100,0%	100,0%
Kendall’s tau-c	-0,339	0,045	-7,554	0,000	0,000^c^				
coefficient	value	stand error.	approximate T	p	p Monte Carlo				

**Table 2:** Relationship between media use and perceived loneliness and anxiety symptoms. .

Tested variables			How many hours a day on average do you spend on social media?					Total
			0Less than 1 hour�	1–2 hours	2–4 hours	4–6 hours	Over 6 hours	
Do you feel lonely despite being active on social media?	aNever	N	30	31	17	3	0	81
		%	52,6%	35,2%	31,5%	17,6%	0,0%	36,8%
	aRdom	N	18	28	10	4	0	60
		%	31,6%	31,8%	18,5%	23,5%	0,0%	27,3%
	Sometimes	N	7	20	14	5	2	48
		%	12,3%	22,7%	25,9%	29,4%	50,0%	21,8%
	Often	N	2	9	10	5	1	27
		%	3,5%	10,2%	18,5%	29,4%	25,0%	12,3%
	Always	N	0	0	3	0	1	4
		%	0,0%	0,0%	5,6%	0,0%	25,0%	1,8%
Total		N	57	88	54	17	4	220
		%	100,0%	100,0%	100,0%	100,0%	100,0%	100,0%
Kendall’s tau-b	0,277	0,053	5,116	0,000	0,000c			
coefficient	value	stand error.	approximate T	p	p Monte Carlo			

**Table 3:** Experience of anxiety and distress symptoms in correlation with age of respondents.

Tested variables			Age						Total
			0Under 18 years of age	18–24 years	25–34 years	35–44 years	45–54 years	55 years and over	
Are you experiencing symptoms such as anxiety, restlessness or lowered mood as a result of your social media use?	aNever	N	3	2	21	32	37	21	116
		%	37,5%	16,7%	52,5%	51,6%	56,1%	65,6%	52,7%
	aRdom	N	3	7	7	17	18	4	56
		%	37,5%	58,3%	17,5%	27,4%	27,3%	12,5%	25,5%
	Sometimes	N	1	2	7	8	9	7	34
		%	12,5%	16,7%	17,5%	12,9%	13,6%	21,9%	15,5%
	Often	N	1	0	5	4	2	0	12
		%	12,5%	0,0%	12,5%	6,5%	3,0%	0,0%	5,5%
	Always	N	0	1	0	1	0	0	2
		%	0,0%	8,3%	0,0%	1,6%	0,0%	0,0%	0,9%
Total		N	8	12	40	62	66	32	220
		%	100,0%	100,0%	100,0%	100,0%	100,0%	100,0%	100,0%
Kendall’s tau-c	-0,116	0,049	-2,357	0,018	0,020^c^				
coefficient	value	stand error.	approximate T	p	p Monte Carlo				

**Table 4:** Awareness of the problem of digital loneliness.

Tested variables			Your level of education					Total
			Basic	Medium	Bachelor’s degree	Master’s degree	zDoctoral or higher	
Do you think digital loneliness is a significant problem in society?	aRather not	N	0	6	1	5	0	12
		%	0,0%	7,6%	3,3%	5,1%	0,0%	5,5%
	I have no opinion	N	1	19	9	16	1	46
		%	14,3%	24,1%	30,0%	16,3%	16,7%	20,9%
	Rather yes	N	5	30	10	37	1	83
		%	71,4%	38,0%	33,3%	37,8%	16,7%	37,7%
	Definitely yes	N	1	24	10	40	4	79
		%	14,3%	30,4%	33,3%	40,8%	66,7%	35,9%
Total		N	7	79	30	98	6	220
		%	100,0%	100,0%	100,0%	100,0%	100,0%	100,0%
Kendall’s tau-c	0,110	0,051	2,178	0,029	0,037^c^			
coefficient	value	stand error.	approximate T	p	p Monte Carlo			

## Discussion

The results of the present study confirm that heavy social media use is associated with a number of negative psychological consequences, including feelings of loneliness and increased anxiety symptoms. Younger users, who spend more time online, are particularly vulnerable to such problems, as reflected in the literature (1, 6, 7). Higher levels of education, on the other hand, seem to act as a protective factor against the negative effects of excessive online activity - increasing awareness of risks and fostering healthier digital habits (8).

A review of the literature and research on this topic has shown that there is a link between excessive use of social networking sites and dysfunctional Internet use (9). As noted by J. Kim, R. LaRose and W. Peng, the negative impact especially in this regard may affect single people. According to the researchers, these individuals not only find it difficult to maintain healthy social interactions in their real lives, but also to regulate their Internet use. Increasing problems lead these people to rely more on their favorite online activity. This is their way of reducing problems or escaping from them. Such activity can further isolate such individuals and increase their loneliness (10).

The research presented here is in line with the results obtained by D. Sieberg. On the basis of these, the author concluded that uncontrolled and indiscriminate use of social networks can hinder the reconstruction of relationships with people met in the real world (11).

The present results also correlate with the observations in the work of Abi-Jaoude et al. where it was also shown that younger users who use social media heavily show a higher risk of anxiety and depressive symptoms (12).

Worth citing are the results of a 2017 UK study conducted by the Royal Society for Public Health (RSPH) among just under 1,500 14–24 year olds. The study focused on the most popular social media, which at the time included: Facebook, YouTube, Instagram, Twitter and the instant messaging service, Snapchat. Respondents were asked to rate how each of the aforementioned platforms influenced fourteen issues related to health and wellbeing. The evaluation consisted of describing their feelings after spending a certain amount of time on a particular service/app. The research tool was a survey questionnaire. Based on the results, the researchers created a ranking of the social media studied in terms of their impact on users’ health and wellbeing. They also found that the only social media that could have a positive impact on users’ health and wellbeing was YouTube. The other social media were classified as having a negative impact on users’ health and wellbeing.

In opposition to the research indicating the negative influence of social media are the opinions propounded by M. Pittman and B. Reich. The researchers believe that image-based social media platforms - of which Instagram is one - can positively influence users by increasing their life satisfaction and decreasing their feelings of loneliness (13).

Undoubtedly, a consequence of social media use is the limited amount of time available to people for offline activities. This time may be limited to the extent that an individual may start to neglect other areas of life, e.g. meeting loved ones, physical activity. The results of a study by K.E. Riehm et al. suggest that adolescents who spend more than three hours a day on social media may be at increased risk of mental health problems, especially internalization problems (14).

It should be noted that excessive social media activity is also associated with sleep problems, reduced quality of life and the emergence of depressive symptoms, as shown in other studies (15, 16). In the context of global challenges - such as the COVID-19 pandemic - the problem of social isolation and stress resulting from reduced face-to-face contact takes on particular significance (17). It has also been suggested that heavy use of social media may promote depressive states among young adults (7). Our observations are in line with these observations: surveyed younger users were more likely to report anxiety symptoms and feelings of loneliness, indicating the need for further monitoring of this at-risk group.

In addition to the intensified experience of loneliness, the use of online platforms, which are increasingly a substitute for real-life interpersonal relationships, can also carry other risks for the psychological sphere.

Evidence of a relationship between social media use and feelings of body image dissatisfaction has been presented (18). Some of the recently published reports explicitly indicate that body image deteriorates as the amount of time spent browsing social media increases (19–22). However, the existence of a high risk of bias in the aforementioned studies should be highlighted. In contrast to the presented results, there is a study by Yurtdas - Depboylu et al. in which no significant difference in self-perception was noted in the context of time spent on social media platforms (23).

In contrast, when considering the issue of perceived self-confidence, inconclusive results have been obtained to date. It is noteworthy, however, that in one study, a significant association was found between lowered self-confidence and the duration of users’ exposure to social media - 3801 members were included in this study, conducted over a period of five years (18).

A significant positive correlation of anxiety disorders with social media use has also been confirmed, as was particularly evident during the COVID-19 pandemic. These disorders arose primarily from comparing self-image with photos depicting the lifestyles of other users (24).

The impact of the aforementioned platforms on the risk of eating disorders is not insignificant, with compulsive overeating syndrome coming to the fore. This thesis seems to be supported by the findings of the work by Livet et al. and Muzi et al. These apply both during and beyond the COVID-19 pandemic (24).

Despite the fact that the vast majority of sources report experiencing long-term loneliness as a major overarching risk factor for mental disorders among children and adults, associations of loneliness with social media use have repeatedly not been demonstrated. Nor have associations been proven with the gender of study participants, their age or previous history of mental illness (25).

In relation to the observations made, it is worth citing the work by Sun et al. They set out to assess the association of social platforms with the intensity of social anxiety, feelings of loneliness and psychological well-being among university students (26). As it turns out, users who use them longer during the day are significantly more likely to have higher symptoms of social anxiety, as shown in a group of students from China (27, 28). Slightly more heterogeneous results are obtained from the point of view of assessing psychological well-being, as roughly equal frequency of use can carry negative as well as beneficial effects. In the near future, it seems necessary to carry out further large-scale analyses to refine the results obtained.

Looking at what has been published to date, it can be concluded that, although the availability of work that considers the elderly group (i.e. those aged 65 and older) is indeed scarce, there are sources that indicate that, in this group, the use of social media can result in a reduction in feelings of social isolation and loneliness, thereby contributing to improved psychological well-being. A meta-analysis by Kusumota et al. considered the results of 11 studies conducted (29). It demonstrated that perceptions of loneliness and isolation can be effectively reduced through the use of digital social media. In addition, the internet can foster greater contact between older people and family members. It can also serve as a source of support, provide a greater sense of community and reduce loneliness (29). However, it seems indispensable to plan further research, taking into account the age groups of older people, in order to verify the current conclusions.

The study conducted is consistent with the reports of many previous works and, at the same time, adds to existing knowledge with new information derived from the clinical group studied. In particular, it seems important to confirm that the phenomenon of digital loneliness may play a significant role as a mediator between excessive social media use and mental health. In other words, the feeling of isolation resulting from online activity may be one of the mechanisms leading to deterioration of mental wellbeing including sleep problems, eating disorders and reduced self-esteem. This fact, combined with the observed differences by age and education, suggests the need to adapt prevention strategies to the specificities of different user groups. Preventive measures are indicated: digital education, parental control, age restrictions and promotion of self-regulation of time spent online. Although their effectiveness has not been studied, they represent an important direction for further analysis.

The results may provide a starting point for the development of prevention programmes in child and adolescent psychiatry, as well as a basis for the development of new diagnostic tools measuring levels of digital loneliness. The study also highlights the need to include online activity in the routine assessment of patients reporting symptoms of mood and anxiety disorders.

## Conclusions

Social media leads to an increased sense of isolation. Excessive use of these platforms is associated with decreased quality of sleep, decreased self-esteem, increased levels of stress and anxiety and an overall deterioration in users’ mental health. The experience of digital loneliness varies according to the characteristics of users - young people, women and people with limitations are the most vulnerable to the negative effects of excessive social media use.

## Data Availability

The original contributions presented in the study are included in the article/supplementary material. Further inquiries can be directed to the corresponding author/s.

## References

[1] Abi-Jaoude E Naylor KT Pignatiello A. Smartphones, social media use and youth mental health. Can Med Assoc J. (2020) 192:E136–41. doi: 10.1503/cmaj.190434, PMID: 32041697 PMC7012622

[2] Blazer D. Social isolation and loneliness in older adults - A mental health/public health challenge. JAMA Psychiatry. (2020) 77:990–1. doi: 10.1001/jamapsychiatry.2020.1054, PMID: 32492078

[3] Al Ahbabi A Al Teneiji A Al Shaer A Al Jaberi G Elsayary A. The effects of social media on self-esteem. In: Proceedings of the 28th World Multi-Conference on Systemics, Cybernetics and Informatics. Florida, USA: WMSCI (2024). p. 185–92.

[4] Błachnio A Przepiorka A Benvenuti M Cannata D Ciobanu AM Senol-Durak E. Cultural and personality predictors of Facebook intrusion. Front Psychol. (2016) 7:1895. doi: 10.3389/fpsyg.2016.01895, PMID: 27994566 PMC5134356

[5] Cichocki Ł Karbownik M. New technologies and mental health - opportunities and difficulties. Psychiatry. (2020) 17:41–8. doi: 10.5603/PSYCH.2020.0007

[6] Hańczuk M Rybołowicz G Szwed J Wilczyńska J Olszewska AM. Impact of social media on interpersonal relationships. Acad Management. (2024) 8:292–313. doi: 10.24427/az-2024-0026

[7] Świdziński R Gryc A Golemo J Nowińska A Lipiec J. Social media and the possibility of depressive states in young adults. J Education Health Sport. (2022) 12:104–12. doi: 10.12775/JEHS.2022.12.12.016

[8] Zsido AN Arato N Lang A Labadi B Stecina D Bandi SA. The role of maladaptive cognitive emotion regulation strategies and social anxiety in problematic smartphone and social media use. Pers Individ Differences. (2021) 173:110647. doi: 10.1016/j.paid.2021.110647

[9] Caplan SE. Preference for online social interaction A theory of problematic internet use and psychosocial well-being, communication research. Sage J. (2023) 30:s. 625. doi: 10.1177/009365020325784

[10] Kim J LaRose R Peng W. Loneliness as the cause and the effect of problematic Internet use: the relationship between Internet use and psychological well-being. CyberPsychology Behavior. (2009) 12:s. 454–455. doi: 10.1089/cpb.2008.0327, PMID: 19514821

[11] Sieberg D. The Digital Diet: The 4-step plan to break your tech addiction and regain balance in your life. Paperback - USA Crown Publishing Group (2011). p. 254. J. Nadobnik, Promoting outdoor activities using selected mobile devices and applications. Częstochowa, Poland: Prace Naukowe Akademii im. Jana Długosza w Częstochowie Kultura Fizyczna. vol. 17, no. 2, p. 146.

[12] Abi-Jaoude E Naylor KT Pignatiello A. Smartphones, social media use and adolescent mental health. CMAJ. (2020) 192:E136–41. doi: 10.1503/cmaj.190434, PMID: 32041697 PMC7012622

[13] Pittman M Reich B. Social media and loneliness: why an Instagram picture may be worth more than a thousand Twitter words. Comput Hum Behavior. (2016) 62:s. 164. doi: 10.1016/j.chb.2016.03.084

[14] Riehm KE Feder KA Tormohlen KN Crum RM Young AS Green KM. Associations between time spent using social media and internalizing and externalizing problems among US youth. JAMA Psychiatry. (2019) 76:s. 1266. doi: 10.1001/jamapsychiatry.2019.2325, PMID: 31509167 PMC6739732

[15] Kolhar M Kazi RNA Alameen A. Effect of social media use on learning, social interactions, and sleep duration among university students. Saudi J Biol Sci. (2021) 28:2216–22. doi: 10.1016/j.sjbs.2021.01.010, PMID: 33911938 PMC8071811

[16] Madrid-Valero JJ Matthews T Barclay NL Odgers CL Moffitt TE Caspi A. Problematic technology use and sleep quality in young adulthood. Sleep. (2023) 46:1–9. doi: 10.1093/sleep/zsad038, PMID: 37106487 PMC10262182

[17] Pyżalski J. Mental health and well-being of young people during the COVID-19 pandemic - a review of the most relevant issues. Child Abuse. (2021) 20:92-115.

[18] Blanchard L Conway-Moore K Aguiar A Önal F Rutter H Helleve A. Associations between social media, adolescent mental health, and diet: A systematic review. Obes Rev. (2023) 24 Suppl 2:e13631. doi: 10.1111/obr.13631, PMID: 37753597

[19] Hosokawa R Kawabe K Nakachi K Soga J Horiuchi F Ueno SI. Effects of social media on body dissatisfaction in junior high school girls in Japan. Eat Behav. (2023) 48:101685. doi: 10.1016/j.eatbeh.2022.101685, PMID: 36512901

[20] Caner N Efe YS Başdaş Ö. The contribution of social media addiction to adolescent LIFE: Social appearance anxiety. Curr Psychol. (2022) 41:8424–33. doi: 10.1007/s12144-022-03280-y, PMID: 35693841 PMC9169592

[21] McAndrew A. What do you see when you look at me? In: Social media, socialized gender variables, and disordered eating among adolescent girls. Doctoral dissertation, University of Windsor, Canada (2020).

[22] Luo YJ Niu GF Kong FC Chen H. Online interpersonal sexual objectification experiences and Chinese adolescent girls’ intuitive eating: the role of broad conceptualization of beauty and body appreciation. Eat Behav. (2019) 33:55–60. doi: 10.1016/j.eatbeh.2019.03.004, PMID: 30927695

[23] Yurtdaş-Depboylu G Kaner G Özçakal S. The association between social media addiction and orthorexia nervosa, eating attitudes, and body image among adolescents. Eat Weight Disord. (2022) 27:3725–35. doi: 10.1007/s40519-022-01521-4, PMID: 36562891

[24] Muzi S Sansò A Pace CS. What’s happened to Italian adolescents during the COVID-19 pandemic? A preliminary study on symptoms, problematic social media usage, and attachment: relationships and differences with pre-pandemic peers. Front Psychiatry. (2021) 12:590543. doi: 10.3389/fpsyt.2021.590543, PMID: 33986698 PMC8110826

[25] Schwartz-Lifshitz M Hertz-Palmor N Dekel I Balan-Moshe L Mekori-Domachevsky E Weisman H. Loneliness and social media use among adolescents with psychiatric disorders. Cyberpsychol Behav Soc Netw. (2022) 25:392–7. doi: 10.1089/cyber.2021.0337, PMID: 35639416

[26] Sun L. Social media usage and students’ social anxiety, loneliness and well-being: does digital mindfulness-based intervention effectively work? BMC Psychol. (2023) 11:362. doi: 10.1186/s40359-023-01398-7, PMID: 37904182 PMC10617103

[27] Schønning V Hjetland GJ Aarø LE Skogen JC. Social media use and mental health and well-being among adolescents - A scoping review. Front Psychol. (2020) 11:1949. doi: 10.3389/fpsyg.2020.01949, PMID: 32922333 PMC7457037

[28] Zhang Y Wang F Cui G Qu J Cheng Y. When and why proactive employees get promoted: A trait activation perspective. Curr Psychol. (2022) 28:1–12. doi: 10.1007/s12144-022-04142-3, PMID: 36590012 PMC9794477

[29] Kusumota L Diniz MAA Ribeiro RM Silva ILCD Figueira ALG Rodrigues FR. Impact of digital social media on the perception of loneliness and social isolation in older adults. Rev Lat Am Enfermagem. (2022) 30:e3573. doi: 10.1590/1518-8345.5641.3573, PMID: 35613252 PMC9132132

